# Interprofessional undergraduate breastfeeding education: A scoping review protocol

**DOI:** 10.12688/hrbopenres.14018.1

**Published:** 2025-01-22

**Authors:** Elaine Lehane, Edina Hanley, Aoife Fleming, Helen Mulcahy, Siobhan Ward, Liz Cogan, Margaret Murphy, Aoife Long, Patricia Leahy-Warren

**Affiliations:** 1 School of Nursing and Midwifery, University College Cork, Cork, County Cork, Ireland; 2 Pharmaceutical Care Research Group, School of Pharmacy, University College Cork, Cork, County Cork, Ireland; 3 Pharmacy Department, Grenville Place, Mercy University Hospital, Cork, Ireland; 4 La Leche League International, Schaumburg, Illinois, USA; 5 University College Cork, Cork, Ireland

**Keywords:** breastfeeding curriculum; health students; interprofessional education

## Abstract

**Objective:**

To review and summarise interprofessional breastfeeding curricula—educational initiatives involving multiple health professions—that have been proposed for undergraduate or pre-registration health students. This review will help guide the development of future Interprofessional Education (IPE) curricula for undergraduate health students, specifically in the area of breastfeeding care.

**Introduction:**

Breastfeeding care and support from healthcare professionals are vital for breastfeeding success. To ensure mothers receive high-quality, consistent care, healthcare professionals must receive comprehensive, evidence-based breastfeeding education. However, there is limited understanding of how breastfeeding curricula are delivered across different disciplines in undergraduate health programs, particularly in the context of IPE.

**Inclusion criteria:**

Primary research designs, including quantitative, qualitative and mixed-method studies and evidence syntheses of primary research including systematic and scoping reviews that meet the inclusion criteria will be considered. Position papers and policy documents will also be considered for inclusion in this scoping review.

**Methods:**

Medline (Ovid), CINAHL (EBSCO), ERIC (EBSCO), Social Sciences, and Cochrane Databases of Systematic Reviews will be searched with English language and date restrictions (2005-current). Titles and abstracts and full-text articles will be independently screened by two reviewers. The reference lists of the included studies will be searched. A grey literature search will be undertaken on Google scholar, BASE and National Institute for Health and Care Excellence (NICE) website in October 2024. Studies will be screened in Covidence by two independent reviewers. All reviewers will agree on the included studies. Data will be extracted and presented graphically using figures and tables. Narrative summary text will accompany the tables and figures.

## Introduction

The World Health Organisation (WHO) recommend exclusive breastfeeding for the first six months of infants’ lives to attain optimal development, growth and health ( WHO, 2023). After six months, it is recommended to continue breastfeeding whilst introducing nutritional, complementary foods for up to two years or beyond ( WHO, 2023). Global breastfeeding rates remain lower than required ( Mulcahy *et al.*, 2022), with approximately 44% of infants between zero to six months breastfed exclusively between 2015–2020 ( WHO, 2023).

Mothers need support to breastfeed, and healthcare professionals play a key role in providing that support ( McFadden *et al.*, 2017; WHO, 2023). However, their breastfeeding knowledge can vary, with gaps reported in the literature ( Leviniene *et al.*, 2009; Mulcahy *et al.*, 2022; Whelan *et al.*, 2011). Comprehensive, evidence-based breastfeeding education during undergraduate training is essential to ensure consistent, high-quality care ( Campbell *et al.*, 2022; UNICEF, WHO, 2003). Yet, curricula often lack sufficient breastfeeding education, leading to varying levels of support for parents ( Campbell *et al.*, 2022; Campbell *et al.*, 2024; Gary *et al.*, 2017).

IPE is crucial for effective healthcare practice implementation ( Homeyer *et al.*, 2018). Learning alongside other disciplines helps students understand both their own roles and the roles of other healthcare professionals ( Saragih *et al.*, 2023). Mulcahy *et al.* (2022) emphasised that interdisciplinary education can improve breastfeeding support. Incorporating an IPE model into healthcare education may foster collaboration and improve students’ knowledge ( Saragih *et al.*, 2023). Applying this IPE approach to breastfeeding curricula could strengthen teamwork among healthcare professionals and improve breastfeeding knowledge. However, there is limited research on how breastfeeding education is integrated across different disciplines in undergraduate health programs. Therefore, this review aims to identify and summarise interprofessional breastfeeding curricula—educational initiatives involving multiple health professions—that have been proposed for undergraduate or pre-registration health students. For this reason, a scoping review will be undertaken.

A preliminary scoping search was undertaken to identify any existing systematic reviews or scoping reviews as per the JBI guidance for scoping reviews ( Peters *et al.*, 2020). Campbell *et al.* (2022) undertook a scoping review of the literature to explore educational resources, methods and curriculum provided to undergraduate health students on lactation. The authors’ recommended the need for a comprehensive breastfeeding-related curriculum for undergraduate health students as approaches to breastfeeding education lack consistency ( Campbell *et al.*, 2022). The current review aims to investigate interprofessional breastfeeding curricula only to inform future IPE curricula for undergraduate health students. 

### Objective

To review and summarise interprofessional breastfeeding curricula (education initiatives that include more than one profession) that have been proposed for undergraduate or pre-registration health students for breastfeeding care to inform future IPE curricula for undergraduate health students.

## Review question

1. What evidence exists regarding IPE on breastfeeding, including the curricular content and intended learning outcomes?
2. How has breastfeeding education been integrated into health professional curricula?
3. What IPE competency frameworks, approaches, principles, or models have been applied in breastfeeding education?
4. Which groups of students have received interprofessional breastfeeding education?
5. What outcomes have been reported in relation to IPE in the context of breastfeeding care?

## Inclusion criteria

### Participants

Undergraduate or pre-registration health students, from any health profession (e.g. dentists, dieticians, doctors, midwives, nurses, occupational therapists, pharmacists, physiotherapists, public health students, speech and language therapists). There will be no restriction on age or gender.

### Concept

Studies that report on breastfeeding curricular content, curricular learning outcomes (skills, attitudes, knowledge, confidence), curriculum integration processes that have been offered to students from two or more professions (i.e. with an IPE component). IPE is defined as education that occurs when “ *students from two or more professions learn about, from, and with each other to enable effective collaboration and improve health outcomes*” ( World Health Organization, 2010)

### Context

Breastfeeding education programmes that include breastfeeding care in the curriculum in Higher Education Institutions (HEI). There will be no limit on countries.

### Types of sources

This scoping review will consider quantitative, qualitative and mixed-method studies. In addition, systematic reviews and scoping reviews that meet the inclusion criteria will also be considered. Position papers and policy documents will also be considered for inclusion in this scoping review.

### Exclusion criteria

Non-health students will be excluded. Studies examining or exploring undergraduate or pre-registration health students’ breastfeeding knowledge, skills, confidence or attitudes without including breastfeeding education/curriculum will be excluded. Studies that include breastfeeding education/curriculum offered to students from only one profession will be excluded. Studies identifying scales that have been developed to assess knowledge acquired during training will be excluded.

## Methods

The Joanna Briggs Institute (JBI) guidelines for scoping reviews will be followed ( Peters *et al.*, 2020). This scoping review will be reported in accordance with the Preferred Reporting Items for Systematic Reviews (PRISMA) extension for scoping reviews, PRISMA-ScR ( Tricco *et al.*, 2018).

### Search strategy

The search strategy will aim to locate both published and unpublished studies. A three-step search strategy as per the JBI methodology for scoping reviews will be utilised in this review ( Peters *et al.*, 2020). First an initial limited search of MEDLINE (Ovid) and CINAHL (EBSCO) was undertaken in September 2024 to identify articles on the topic. The text words contained in the titles and abstracts of relevant articles were used to develop a full search strategy for Medline (Ovid), CINAHL (EBSCO), ERIC (EBSCO), Social Sciences and Cochrane Databases of Systematic Reviews ( Table 1 and Table 2). Keywords were mapped to relevant subject headings for the appropriate databases. The search strategy was reviewed by topic experts and knowledge users who are professionals in this area.

**Table 1.** Developing the Search Strategy.

Search concepts	Breastfeeding/Breastfeeding Support	Curriculum/Education	Undergraduate/Health Student
**Search terms**	Breastfeeding, Breast-feeding, Breastfeed, Breast-feed, Breastfed, Breast-fed, Lactation, Infant feed, Infant Feeding, Infant-fed, Breastfeeding support, Breastfeeding promotion	Course, Curriculum, curricular, curricula, education, educate, teaching, teach, training, train, learn, learning, program, knowledge, skill, information, instruct, intervention, interprofessional, interdisciplinary, interprofessional learning, interprofessional education	Student, Trainee, Undergraduate, Baccalaureate, Pre-licensure, Novice, Pre-qualification, Pre-certification, Pre-professional, Health student, healthcare student, health professional student, student nurse, nursing student, student midwife, student midwives, midwifery student, nurse midwifery student, medical student, medicine student, pharmacy student, student pharmacist, public health student, dentistry student, dental student, student dentist, speech-language pathology student, speech pathology student, speech-language therapy student, speech therapy student, SLT/SLP students, OT student, occupational therapy student, physiotherapy student, physical therapy students, Student dietitian, student dietician, student nutritionist

**Table 2.** Search Strategy.

Search	Query
**#1**	**Title/Abstract:** Breastfeed* OR “breast-feed*” OR Breastfed OR “Breast-fed” OR Lacta* OR “infant feed*” OR “infant- feed*” OR “infant-fed” OR “Breastfeeding support” OR “Breastfeeding promotion”
**#2**	**Title/Abstract:** Course OR Curricul* OR educat* OR teach* OR train* OR learn* OR program* OR knowledge* OR skill* OR information OR instruct* OR intervention OR interprofessional* OR interdisciplinary OR "interprofessional* education" OR "interprofessional educat*" OR “interprofessional* learning” OR “interprofessional learn*”
**#3**	**Title/Abstract:** Student* OR Trainee* OR Undergraduate* OR Baccalaureate* OR “Pre-licensure” OR Novice* OR “Pre-qualification” OR “Pre-certification” OR “Pre-professional*” OR “health student*” OR “healthcare student*” OR “health care student*” OR “health professional student*” OR “nursing student*” OR “student nurs*” OR “nurse midwifery student*” OR “midwife student*” OR “student midwi*” OR “midwifery student*” OR “medical student*” OR “medicine student*” OR "medic* student" OR “pharmacy student*” OR “student pharmacist*” OR “occupational therap* student” OR “occupational therapy student*” OR “OT student*” OR “speech pathology student*” OR “speech language therapy student*” OR “speech language therap* student” OR “speech language patholog* student" OR “speech therap* student” OR “speech patholog* student” OR “speech language pathology student*” OR “SLT student*” OR “SLP student*” OR "physiotherapy student*” OR “physical therapy student*" OR "physiotherap* student" OR "physical therap* student" or "dentistry student*" or "dental student*" OR "dentist* student" OR "nutrition* student" OR "Student* Dietitian" OR "Student* Dietician" OR "Student* nutritionist" OR "public health student*”
**#4**	#1 AND #2 AND #3
**Limits**	English language, 2005-current

The search strategy will be adapted for each included database. Syntax and subject headings will be adapted to match each database. One full search has been included in the protocol ( Table 3). The reference list of all included studies will be searched to identify additional studies. A grey literature search will be undertaken on Google scholar, BASE and NICE website. Studies published in English will be included. The date range of evidence will be set to 2005 to the current time. This parameter was set in line with international recommendations for curriculum content in breastfeeding support to be included in undergraduate programs (The Global Strategy for Infant and Young Child Feeding, 2003; The NICE Guidelines on Maternal and Infant Nutrition, 2008;2014).

**Table 3.** Developing the Search Strategy Search Strategy CINAHL (EBSCO Searched September 2024).

Search	Query	Records retrieved
**#1**	**CINAHL Subject Headings:** Infant feeding OR Breast feeding OR Lactation **OR** **Title/Abstract:** Breastfeed* OR “breast-feed*” OR Breastfed OR “Breast-fed” OR Lacta* OR “infant feed*” OR “infant-feed*” OR “infant-fed” OR “Breastfeeding support” OR “Breastfeeding promotion”	**67, 773**
**#2**	**CINAHL Subject Headings:** Curriculum OR Education OR "Education, Interdisciplinary" OR learning OR teaching OR knowledge **OR** **Title/Abstract:** Course OR Curricul* OR educat* OR teach* OR train* OR learn* OR program* OR knowledge* OR skill* OR information OR instruct* OR intervention OR interprofessional* OR interdisciplinary OR "interprofessional* education" OR "interprofessional educat*" OR “interprofessional* learning” OR “interprofessional learn*”	**2, 006, 797**
**#3**	**CINAHL Subject Headings:** Students, Undergraduate OR Students, nurse midwifery OR Students, nursing OR Students, nursing, baccalaureate OR Students, midwifery OR Students, pharmacy OR Students, medical OR Students, dental OR Students, occupational therapy OR Students, speech-language pathology OR Students, physical therapy OR Students, Dietetics OR Students, health occupations **OR** **Title/Abstract:** Student* OR Trainee* OR Undergraduate* OR Baccalaureate* OR “Pre-licensure” OR Novice* OR “Pre-qualification” OR “Pre-certification” OR “Pre-professional*” OR “health student*” OR “healthcare student*” OR “health care student*” OR “health professional student*” OR “nursing student*” OR “student nurs*” OR “nurse midwifery student*” OR “midwife student*” OR “student midwi*” OR “midwifery student*” OR “medical student*” OR “medicine student*” OR "medic* student" OR “pharmacy student*” OR “student pharmacist*” OR “occupational therap* student” OR “occupational therapy student*” OR “OT student*” OR “speech pathology student*” OR “speech language therapy student*” OR “speech language therap* student” OR “speech language patholog* student" OR “speech therap* student” OR “speech patholog* student” OR “speech language pathology student*” OR “SLT student*” OR “SLP student*” OR "physiotherapy student*” OR “physical therapy student*" or "physiotherap* student" OR "physical therap* student" OR "dentistry student*" OR "dental student*" OR "dentist* student" OR "nutrition* student" OR "Student* Dietitian" OR "Student* Dietician" OR "Student* nutritionist" OR "public health student*”	**265,212**
**#4**	#1 AND #2 AND #3	**534**
**Limits**	English language, 2005-current	**445**

### Study/Source of evidence selection

Following the search, all identified citations will be transferred to Covidence, and duplicates removed. Following a pilot test by two independent reviewers, titles and abstracts will then be screened by two independent reviewers for assessment against the inclusion criteria for the review. Potentially relevant sources will be retrieved in full and screened in Covidence. The full text of selected citations will be assessed in detail against the inclusion criteria by two independent reviewers. Any disagreements that arise between the reviewers at each stage of the selection process will be resolved through discussion. Where consensus cannot be reached, a third reviewer will be consulted ( Peters *et al.*, 2020). Reasons for exclusion of studies will be provided. The results of the search and the study inclusion process will be reported in the final scoping review and presented in a PRISMA flow diagram ( Tricco *et al.*, 2018). 

### Data extraction

Data will be extracted from papers included in the scoping review by two independent reviewers using a data extraction template ( Peters *et al.*, 2020) that will be adapted by the reviewers ( Table 4). The JBI data extraction template was modified to include the PCC relevant to this review. Data relevant to address the review objectives and associated questions will be extracted ( Peters *et al.*, 2020). The following data will be extracted; author, year, country, type of evidence source, study aim and objectives, participants, context, methodology, curricular content, learning outcomes, integration processes, IPE components, delivery approach, teaching and assessment strategy and key findings as they relate to the review questions.

**Table 4.** Data extraction instrument.

Author, Year, Country	Type of evidence source	Study aim/ objective	1.Methodology 2.Participants 3.Context	1. Curricular Content 2.Learning Outcomes 3.Integration Processes	IPE Component (e.g. Competency framework, collaboration)	1.Delivery Approach 2.Teaching and Assessment Strategy	Key Findings
							

The data extraction form will be piloted by two reviewers independently on two studies before use ( Peters *et al.*, 2020). Revisions will be made as required. The draft data extraction tool will be modified and revised as necessary during the process of extracting data from each included evidence source. Modifications will be detailed in the scoping review. Any disagreements that arise between the reviewers will be resolved through discussion, or with a third reviewer. If appropriate, authors of papers will be contacted to request missing or additional data, where required.

### Data analysis and presentation

The evidence will be mapped and summarised in line with the review aims and objectives. The data will be presented graphically using figures and tables. Narrative summaries will accompany these results. The included studies will be summarised based on the studies’ characteristics, including the year, country and type of evidence source, study aim and objectives, participants, context and methodology. Narrative summaries will outline characteristics of interprofessional breastfeeding curricula, including curricular content, learning outcomes, integration processes, delivery approach, teaching and assessment strategy, IPE approaches, competency frameworks, models or principles and key findings as they relate to the review questions. This information will be analysed to inform future IPE curricula for undergraduate health students.

A reflexive approach will be adopted throughout the review process. The reviewers will critically reflect on the selection of studies, interpretation of the literature and the potential biases inherent in the synthesis process. Reflexivity will involve the reviewers’ being transparent about their perspectives, decisions made during the review process, and how these may influence findings. A reflexive statement will be included in the review discussing these considerations to provide a richer understanding of the choices made and how these may shape the conclusions drawn.

## Ethics and consent

Ethics and consent were not required

## Data Availability

No data are associated with this article.
