# Supplementary material for: Interprofessional undergraduate breastfeeding education: A scoping review protocol
Source: HRB Open Res. 2025 Jan 22;8:6. [Version 1] doi: 10.12688/hrbopenres.14018.1 (PMC11937782; doi:10.12688/hrbopenres.14018.1)
Supplement: Peer review history

# Reviewer report: Reviewer response for version 1

**DOI:** 10.21956/hrbopenres.15387.r44916

**Published:** 2025-03-25

**Authors:** Claire O'Donnell

**Affiliations:** 1 University of Limerick, Limerick, County Limerick, Ireland

This protocol is a timely review of educational breastfeeding initiatives of healthcare professionals proposed for undergraduate/pre-registration health students. The robust search strategy and wide sampling population are a strength of review. Clear rationale provided and all steps/processes of this scoping review are clearly detailed in line with JBI criteria. Use of Covidence in data management supports replication of study by others. Inclusion of reflexivity is commended in supporting transparency. This review is a welcomed piece of work in supporting mothers with breastfeeding.

Is the study design appropriate for the research question?

Yes

Is the rationale for, and objectives of, the study clearly described?

Yes

Are sufficient details of the methods provided to allow replication by others?

Yes

Are the datasets clearly presented in a useable and accessible format?

Yes

Reviewer Expertise:

Expertise in the area of scoping reviews and also breastfeeding

I confirm that I have read this submission and believe that I have an appropriate level of expertise to confirm that it is of an acceptable scientific standard.

# Reviewer report: Reviewer response for version 1

**DOI:** 10.21956/hrbopenres.15387.r46163

**Published:** 2025-03-05

**Authors:** Antonio Jesús Ramos-Morcillo

**Affiliations:** 1 University of Murcia, Murcia, Spain

The rationale for the study is well-articulated. The authors clearly explain the importance of breastfeeding education, the role of healthcare professionals, and the lack of interprofessional breastfeeding curricula. The objectives are explicitly stated, aiming to identify and summarize interprofessional breastfeeding education initiatives.

 A scoping review is a suitable approach for mapping available literature on interprofessional breastfeeding education. The study follows established guidelines (JBI and PRISMA-ScR), ensuring a systematic and replicable process. The methodology is appropriate for addressing the research question, which focuses on the breadth and nature of existing educational programs rather than effectiveness alone.

 The methodology is described in detail, including the search strategy, inclusion and exclusion criteria, databases consulted, and the process of data extraction. The authors also specify the screening process, involving two independent reviewers, with mechanisms to resolve disagreements. These elements ensure that the study could be replicated by others following the same methodology.

 Inclusion criteria. I do not know why speech and language therapist professionals should be included.

Is the study design appropriate for the research question?

Yes

Is the rationale for, and objectives of, the study clearly described?

Yes

Are sufficient details of the methods provided to allow replication by others?

Yes

Are the datasets clearly presented in a useable and accessible format?

Not applicable

Reviewer Expertise:

My area of expertise is implementation of clinical practice guidelines, breastfeeding, evidence-based practice, primary health care.

I confirm that I have read this submission and believe that I have an appropriate level of expertise to confirm that it is of an acceptable scientific standard.

# Reviewer report: Reviewer response for version 1

**DOI:** 10.21956/hrbopenres.15387.r45157

**Published:** 2025-02-10

**Authors:** Oluwatosin Leshi

**Affiliations:** 1 University of Kentucky, Lexington, Kentucky, USA

1. The emphasis in the opening paragraph was on exclusive breastfeeding, however, the scope of the study goes beyond exclusive breastfeeding but the concept of breastfeeding as a whole. The author might want to consider opening the paragraph with global sub-optimal breastfeeding 

 2. In the last sentence of paragraph 2, the authors need to specify the type of curricula whether college/university curriculum or breastfeeding education curriculum at hospitals.

 3. It is a recommended practice that review protocols are pre-registered to ensure quality outputs. The authors might need to indicate which registration platform was used and possibly the registration number for the protocol.

Is the study design appropriate for the research question?

Yes

Is the rationale for, and objectives of, the study clearly described?

Yes

Are sufficient details of the methods provided to allow replication by others?

Yes

Are the datasets clearly presented in a useable and accessible format?

Yes

Reviewer Expertise:

Maternal and Child Health, Health Disparity, Community-Engaged Research

I confirm that I have read this submission and believe that I have an appropriate level of expertise to confirm that it is of an acceptable scientific standard, however I have significant reservations, as outlined above.
